# Impact of a high dietary fiber cereal meal intervention on body weight, adipose distribution, and cardiovascular risk among individuals with type 2 diabetes

**DOI:** 10.3389/fendo.2023.1283626

**Published:** 2023-10-30

**Authors:** Xiaofeng Li, Yu Shi, Dongqin Wei, Wenyu Ni, Na Zhu, Xinyi Yan

**Affiliations:** ^1^ Department of Endocrinology, Metabolic Management Center, Qidong People’s Hospital, Qidong Liver Cancer Institute, Affiliated Qidong Hospital of Nantong University, Jiangsu, China; ^2^ Department of Remote ECG Diagnostic Center, Qidong People’s Hospital, Qidong Liver Cancer Institute, Affiliated Qidong Hospital of Nantong University, Jiangsu, China

**Keywords:** cereal-based nutritional intervention, overweight, type 2 diabetes, cardiovascular risk, metabolic syndrome

## Abstract

**Objective:**

This study sought to examine the impacts of a high dietary fiber cereal meal in comparison to conventional dietary management for diabetes on body weight, distribution of adipose tissue, and cardiovascular risk among individuals diagnosed with type 2 diabetes (T2DM).

**Methods:**

A cohort of 120 patients diagnosed with T2DM was enlisted as the study population and divided into two groups using a ratio of 2:1—namely, the W group (n=80) and the U group (n=40). The U group (control) received usual diet, while the W group (intervention) incorporated a high dietary fiber cereal meal in place of their regular staple food in addition to adhering to conventional diabetes dietary recommendations. The high dietary fiber cereal meal was based on whole grains, traditional Chinese medicinal foods, and prebiotics. A subsequent follow-up period of 3 months ensued, during which diverse parameters such as body mass index (BMI),waist-hip ratio (WHR), glycated hemoglobin (HbA1c),fasting blood glucose(FBG),C-peptide levels, blood pressure, blood lipids, high-sensitivity C-reactive protein (hsCRP),10-year cardiovascular disease (CVD) risk, and Lifetime CVD risk were assessed before and after the intervention.

**Results:**

Among the participants, a total of 107 successfully completed the intervention and follow-up, including 72 individuals from the W group and 35 from the U group. Following the intervention, both cohorts exhibited decrease in BMI, WHR, HbA1c, FBG, blood pressure, and blood lipid levels in contrast to their initial measurements. Remarkably, the improvements in BMI, WHR, HbA1c, FBG, total cholesterol (TC), triglycerides(TG), low-density lipoprotein cholesterol (LDL-C), the ratio of triglyceride to high-density lipoprotein cholesterol (TG/HDL-C), and the ratio of 2-hour C-peptide (2hCP) to fasting C-peptide (FCP) were more marked within the W group, exhibiting statistically significant disparities (*P*<0.05) in comparison to the U group. Furthermore, the levels of hsCRP declined among individuals in the W group, while the U group experienced an elevation.10-year CVD risk reduction were similar in the two groups. While, Lifetime CVD risk only decreased significantly in the W group.

**Conclusion:**

The intervention centred on a cereal-based dietary approach showcased favourable outcomes with regard to body weight, adipose distribution, and cardiovascular risk in overweight individuals grappling with T2DM.

## 1 Introduction

Cardiovascular disease is the leading cause of death among individuals with diabetes. Diabetes itself is an independent risk factor for cardiovascular disease, increasing the risk by 2 to 4 times compared to those without diabetes. It is worth noting that diabetes often coexists with other significant cardiovascular risk factors, such as hypertension and lipid abnormalities (1). The combination of clinical manifestations, including obesity, hyperglycemia, hypertension, and dyslipidemia, is referred to as metabolic syndrome. This collection of factors significantly contributes to the development of atherosclerotic cardiovascular diseases (ASCVD). Research has shown that among diabetes patients, those with uncontrolled metabolic syndrome components face higher risks of adverse cardiovascular outcomes compared to those with well-managed components (2). Comprehensive interventions that address multiple risk factors have the potential to significantly reduce the incidence and mortality associated with cardiovascular diseases in individuals with diabetes (3). The management of body weight is an essential aspect of type 2 diabetes (T2DM) therapy. Achieving optimal weight not only improves glycemic control and reduces the need for hypoglycemic medications but also leads to improvements in other metabolic parameters among diabetic patients, including blood pressure and lipid profiles.

Obese T2DM patients often exceed their daily caloric intake, primarily attributed to disproportionate carbohydrate consumption (4). A multitude of investigations on ketogenic diets have exhibited that stringent carbohydrate reduction substantively facilitates weight management and heightens the state of metabolic syndrome constituents. However, sustaining steadfast adherence over prolonged periods remains a formidable task for patients (5). Within the context of Chinese diabetic patients’ dietary habits, it is advocated that carbohydrates constitute 50% to 65% of the overall energy intake. For individuals grappling with suboptimal postprandial blood glucose control, a modest curtailment in energy sourced from carbohydrates is deemed advisable. Nevertheless, the enduring adoption of an extremely low-carbohydrate diet is not recommended on a protracted basis (6). The findings from the Atherosclerosis Risk in Communities (ARIC) study underscore that the nadir of mortality risk is attained when carbohydrates contribute to 50% to 55% of the overall energy intake (7). The consumption of whole grains exhibits an inverse correlation with the hazards associated with all-cause mortality, coronary heart disease,T2DM, and colorectal cancer. Similarly, dietary fiber intake manifests a negative correlation with the perils linked to all-cause mortality, coronary heart disease, T2DM, and colorectal cancer (8). Consequently, transcending the mere oversight of total carbohydrate intake, due attention ought to be devoted to the caliber of carbohydrates, with a pronounced focus on augmenting dietary fiber intake. Adults are advised to incorporate over 14 g of dietary fiber per 1,000 kcal daily (9).

In the management of diabetes patients, continuous cardiovascular risk assessment is necessary in order to timely identify individuals at high risk of cardiovascular events, and facilitate the development of personalized treatment plans by clinical doctors. The cardiovascular disease prediction model evaluates the probability of an individual developing cardiovascular disease in the future based on the levels and combinations of multiple cardiovascular risk factors. They can be divided into short-term risk prediction and long-term risk prediction. Short-term risk generally refers to a ten-year risk, while long-term risk generally refers to a risk beyond ten years or lifetime risk. There are various types of cardiovascular disease prediction models. The main cardiovascular risk assessment models in Europe and the United States include the Systematic Coronary Risk Estimation (SCORE) model (10), the QRISK score model in the UK (11, 12), and the Pooled Cohorts Equations (PCE) model (13) for ASCVD risk assessment. Chinese scholars have used large sample cohort data from the Prediction for ASCVD Risk in China (China-PAR) study to establish the China-PAR model for 10-year and lifetime cardiovascular risk assessment (14).

Nutritional therapy has burgeoned as a pivotal stratagem in the prevention and management of diabetes and its complications (15). The dietary intervention chosen for this inquiry, namely the high dietary fiber cereal meal, is rooted in whole grains, traditional Chinese sustenance, and prebiotics(WTP). The crux of this study resides in delving into the repercussions of the high dietary fiber cereal meal on metabolic markers among individuals grappling with T2DM.Use the China-PAR model to assess the impact of this intervention on the cardiovascular disease risk in those patients.

## 2 Materials and methods

### 2.1 Inclusion of patients and general information

During the period spanning March 2015 to April 2015, a comprehensive total of 120 patients who had received a diagnosis of T2DM were enlisted from the outpatient clinic of the Endocrinology Department at our institution. Through employment of a random allocation table, these participants were categorically divided into two distinct groups: the W group (intervention) and the U group (control), adhering to a proportion of 2:1. The inclusive criteria incorporated the following requisites: Adherence to the diabetes diagnostic parameters as stipulated by the World Health Organization (WHO). An age range of 35 to 70 years. The values of HbA1c ranged from 6.0% to 12.0%. Demonstrable compliance, indicated by negligible occurrences of travel, outdoor dining, and consistent adherence to daily routines. Weight stability within a variance of 2 kg over the course of the preceding 3 months. The exclusion criteria encompassed the following conditions: Patients diagnosed with type 1 diabetes. Individuals with intentions of pregnancy, presently pregnant, or engaged in breastfeeding during the study period. Patients afflicted by severe diabetes complications, such as diabetic nephropathy and diabetic foot. Individuals who had experienced acute episodes of mental illness within the preceding 3 months. Patients with recent history of acute occurrences of ailments like cholecystitis, gastrointestinal ulcers, and upper and lower urinary tract infections within the past three months. Uncontrolled hyperthyroidism, adrenal, or pituitary disorders. Individuals who underwent gastrointestinal surgery within the last year, with the exception of appendectomy and hernia surgery. Patients contending with severe liver ailments like chronic hepatitis and cirrhosis, or possessing abnormal liver function (serum alanine amino transferase and aspartate amino transferase levels exceeding 2.5 times the norm). Those with non-specific inflammatory conditions of the intestines. Patients suffering from acute or chronic renal insufficiency. Individuals afflicted with significant cardiovascular conditions such as tumors, coronary heart disease, and stroke. Those with infectious disorders such as tuberculosis and HIV/AIDS. Anemia indicated by hemoglobin levels below 10 g/dL. Individuals who had resorted to medication-based interventions (inclusive of appetite suppressants like fluoxetine, thyroid medications, progesterone, laxatives, and various traditional Chinese weight-loss remedies) or surgical methods for weight loss within the preceding three months. Participants unable to allocate adequate time for project involvement. Systolic blood pressure equal to or exceeding 180 mmHg, or diastolic blood pressure equal to or exceeding 110 mmHg.

The individuals participating in the study, as well as their family members, were adequately informed about the intricacies of the study and accorded their informed consent through signature. The medical ethics committee affiliated with the hospital was apprised of the study’s design and granted their approval for its execution. The trial was registered in the Chinese Clinical Trial Registry (ChiCTR-IPC-14005346).

### 2.2 Methods and implementation

Throughout the entirety of the experimental process, all patients diligently adhered to their existing antihypertensive and lipid-lowering therapeutic regimens. As a measure to uphold patient well-being during the study, endocrinologists cautiously adjusted glucose-lowering medications when patients encountered extreme blood glucose values. Considering that glucagon-like peptide-1 receptor agonists (GLP-1RAs) and sodium-glucose co-transporter-2 (SGLT-2) inhibitors have a more significant impact on patient weight, these two types of medications were not initiated during the follow-up period.

**Figure 1:**
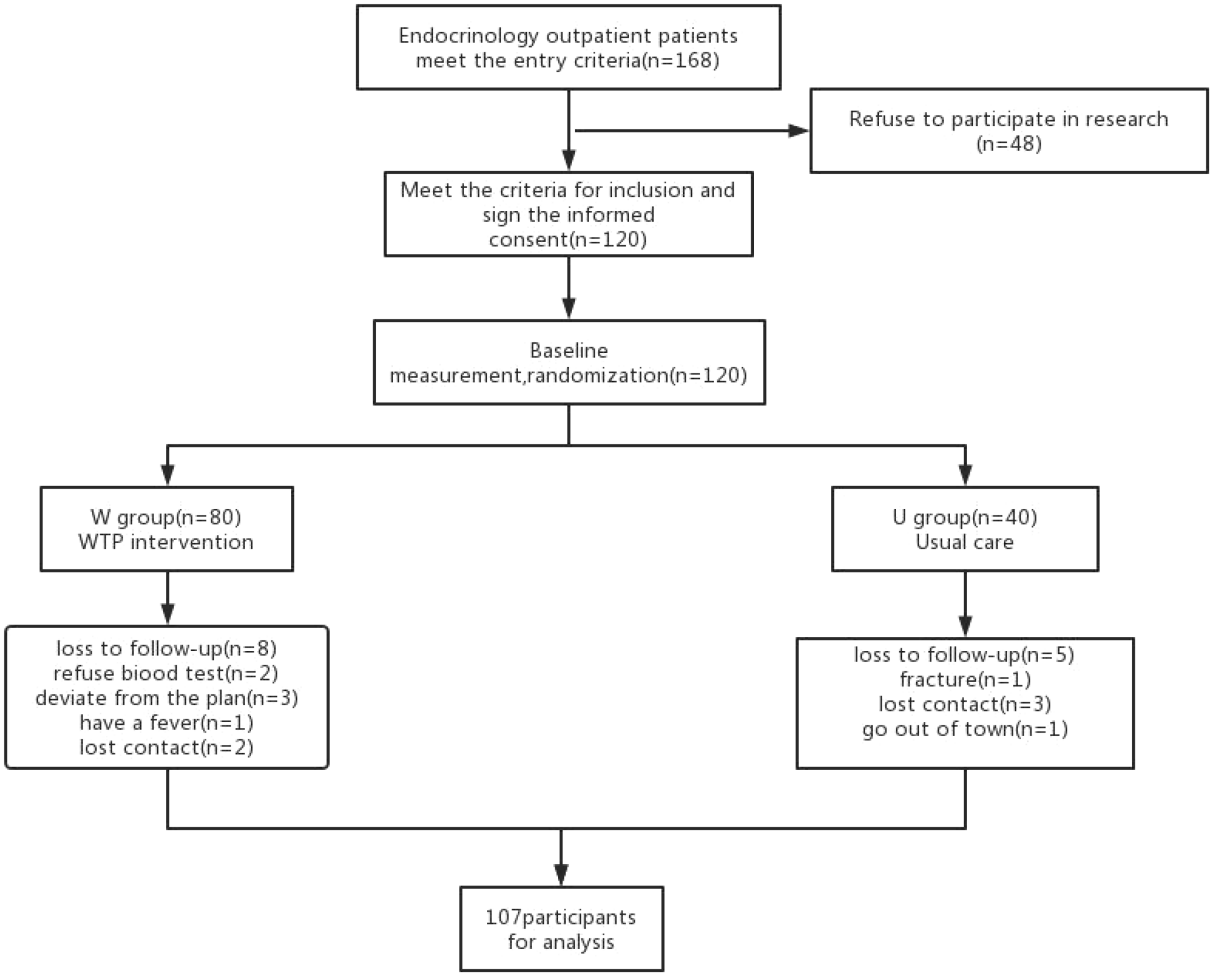
Flow chart of the study population.

Patients assigned to the U group received conventional diabetes dietary guidance, aligned with the tenets outlined in the Chinese Diabetes Dietary Guidelines. Conversely, participants in the W group were furnished with a high dietary fibre cereal meal, intended to supplant their routine daily staple foods. This cereal meal, presented as a prepackaged product, was fashioned from two integral components: pre-packaged porridge (Component 1) and blending agents (Component 2 and 3), provided by Perfect (China) Co., Ltd. Component 1, encompassing the pre-packaged porridge, was formulated through the amalgamation of nine precooked constituents sourced from whole grains and traditional Chinese food crops. Abounding in dietary fibre, its composition encompassed ingredients such as barley, buckwheat, oats, Chinese yam, lentils, red beans, soybeans, corn, red dates, goji berries, lotus seeds, and peanuts. Each canister weighed 360 grams when wet. Component 2, with a content of 10 grams per packet, predominantly featured bitter melon powder and a blend of low-molecular-weight oligosaccharides. Component 3, amounting to 15 grams per packet, contained kudzu starch, inulin, and resistant dextrin. Individuals allocated to the W group were instructed to consume a minimum of 360 grams of pre-packaged porridge from Component 1 during each meal, in conjunction with one packet each of Component 2 and Component 3. In addition, a suitable quantity of vegetables, legumes, and fruits could be integrated as accompaniments. **Table 1** shows the nutritional composition of high dietary fibre cereal meal. **Figure 1** shows flow chart of the study population. Both groups of patients were asked to record their daily diet and medication usage in a logbook. Patients in Group W exchanged empty packaging bags of intervention food at the end of each month for the next month’s intervention food.

**Table 1:** Nutritional composition of high dietary fibre cereal meal.

	Component 1^a^	Component 2^b^	Component 3^b^
Carbohydrates (g/100g)	10.5	61.7	85.7
Protein (g/100g)	2.95	9.76	<0.1
Fat (g/100g)	1.0	1.2	<0.1
Dietary Fiber (g/100g)	1.4	18.9	6.6
Soluble Dietary Fiber (g/100g)	0.4	5.6	6.6
Insoluble Dietary Fiber (g/100g)	1.0	13.3	<0.1
Sodium (mg/kg)	41	1950	102
Potassium (mg/kg)	1370	1920	227

a. Component 1 is a pre-cooked food. The concentration of each nutrient is per 100 g wet weight.

b. Component 2 and 3 are dry powder. The concentration of each nutrient is per100 g dry weight.

## 3 Observation parameters

Prior to and after the 3-month treatment interval, an assortment of vital metrics were meticulously observed and documented. These included the patients’ stature, mass, blood pressure readings, waist circumference(WC), hip circumference, comprehensive blood count, HbA1c levels, FBG, FCP, liver and kidney functionality, blood lipid profiles (TG, TC, LDL-C, HDL-C), oral glucose tolerance test (OGTT) 2hCP, and levels of hsCRP.

The China-PAR risk assessment model includes gender, age, place of residence (urban or rural), region (north or south, with the Yangtze River as the boundary), WC,TC,HDL-C, current blood pressure level, use of antihypertensive medication, presence of diabetes, current smoking status, and family history of cardiovascular disease.

## 4 Sample size

The sample size was calculated based on a previous study conducted by Zhao et al. (16). In Zhao et al.’s study,49 participants were randomly assigned to the intervention or control group in a 2:1 ratio. There was a mean HbA1cdifference of 0.61 mmol/L between the intervention group and the control group.To get 90% of power using a two-tailed hypothesis, the intervention group would require 26 patients. Considering a 20% dropout rate, a sample of 49 patients divided into 2 groups would be necessary in order to detect a difference. The sample size of this study was sufficient.

## 5 Statistical analysis

The statistical analysis was executed using SPSS 22.0 software. Enumeration data were displayed as N/% and subjected to scrutiny through the x2 test. Continuous data were expressed as $\bar{x}\pm s$ and analyzed using the t-test, provided they conformed to a normal distribution. For data that deviated from the normal distribution, changes observed before and after the study period were presented as a median (interquartile range) and subsequently compared across groups using the Wilcoxon rank-sum test. The statistical significance was defined as a *P* -Value of less than 0.05.

## 6 Results

A total of 107 participants successfully completed the intervention and the subsequent follow-up phase, with the W group including 72 individuals and the U group including 35 participants.

### 6.1 The foundational data of the patients

There were no statistically significant differences between the two patient groups in terms of average age, BMI, and glycated hemoglobin levels (*P >*0.05). The proportion of female patients in the W group was slightly higher than that in the U group. Additionally, the W group had slightly higher mean HDL-C levels compared to the U group **Table 2**. All patients followed local dietary habits and consumed relatively stable amounts of carbohydrates, proteins, and fats daily. Based on the patients’ dietary records and the recycling statistics of empty packaging bags of intervention food, the compliance of Group W patients with a high-fiber diet was approximately estimated to be 90%. Patients in Group W reported feeling fuller and experiencing a decrease in appetite compared to before the intervention, while patients in Group U reported no significant changes in appetite before and after the intervention.

**Table 2:** General data of patients.

	W Group (n=72)	U Group (n=35)	*t-*value/Wilcoxon Rank Sum	*P*-Value
Female n (%)	50(69.4%)	21(60%)	0.94 (*χ* ^2^)	0.3320
Mean Age (years)	60.86 ± 7.25	58.51 ± 8.02	1486.00	0.1327
BMI (kg/m^2^)	24.75 ± 4.28	25.40 ± 2.80	1037.00	0.1386
HbA1c (%)	8.15 ± 1.20	8.11 ± 1.20	0.17	0.8678
TC (mmol/L)	5.21 ± 1.57	4.86 ± 1.23	1543.00	0.0602
TG (mmol/L)	1.15 ± 0.71	1.39 ± 0.92	1014.50	0.1030
HDL-C (mmol/L)	1.37 ± 0.31	1.23 ± 0.27	2.34	0.0219^*^
LDL-C (mmol/L)	2.64 ± 1.07	2.63 ± 0.74	1379.00	0.4294
SBP (mmHg)	127.94 ± 14.89	131.54 ± 14.93	-1.17	0.2459
DBP (mmHg)	70.89 ± 10.22	74.26 ± 11.05	-1.52	0.1346

BMI, body mass index; HbA1c, glycated hemoglobin; TC, total cholesterol; TG, triglycerides; HDL-C, high-density lipoprotein cholesterol; LDL-C, low-density lipoprotein cholesterol; SBP, systolic blood pressure; DBP, diastolic blood pressure.

### 6.2 The comparative evaluation of clinical efficacy between the two patient groups

It was observed that the improvement in weight, BMI, waist-hip ratio, glycated hemoglobin, fasting blood glucose, TC, TG, LDL-C, TG/HDL-C, and 2hC-peptide/fasting C-peptide was better in the W group compared to the U group, with statistically significant differences (*P <*0.05). hsCRP decreased in the W group and increased in the U group. There were no significant differences in WC, HDL-C, and blood pressure changes between the two groups **Table 3**.

**Table 3:** Comparison of Metabolic Parameters in T2DM Patients with and without High Dietary Fiber Intervention.

Observation Parameters	Changes Before and After Intervention in Group W	Changes Before and After Intervention in Group U	*t-*value/Wilcoxon Rank Sum	*P*-Value
Body mass (kg)	-2.93 ± 3.24	-1.69 ± 1.23	-2.84	0.0055^*^
BMI (kg/m^2^)	-1.10 ± 1.24	-0.64 ± 0.48	-2.75	0.0071^*^
WC (cm)	-2.38 ± 6.28	-1.65 ± 6.45	-0.56	0.5800
HC (cm)	-3.00 ± 6.00	0.00 ± 3.25	654.00	3.686×10^-5*^
WHR	0.01 ± 0.06	-0.01 ± 0.06	2.04	0.0455^*^
TC (mmol/L)	-0.93 ± 0.93	-0.42 ± 0.72	-3.12	0.0024^*^
TG (mmol/L)	-0.26 ± 0.46	0.02 ± 0.97	900.50	0.0128^*^
HDL-C (mmol/L)	-0.05 ± 0.19	-0.07 ± 0.15	0.46	0.6440
LDL-C (mmol/L)	-0.17 ± 0.61	0.08 ± 0.44	-2.43	0.0172^*^
TG/HDL-C	-0.16 ± 0.44	0.05 ± 0.82	842.50	0.0041^*^
HbA1c (%)	-1.31 ± 0.99	-0.43 ± 1.02	-4.25	6.730×10^-5*^
SBP (mmHg)	-3.30 ± 15.67	-4.69 ± 12.76	0.50	0.6217
DBP (mmHg)	-4.00 ± 11.50	-2.00 ± 8.75	1174.50	0.4945
FBG (mmol/L)	-2.41 ± 2.05	-1.02 ± 2.00	-3.37	0.0012^*^
hsCRP (mg/L)	-0.30 ± 1.10	0.20 ± 1.53	878.00	0.0083^*^
2hCP/FCP	0.54 ± 1.17	-0.06 ± 1.07	-2.52	0.013

BMI, body mass index; WC, waist circumference; HC, hip circumference; WHR, waist–hip ratio; TC, total cholesterol; TG, triglycerides; HDL-C, high-density lipoprotein cholesterol; LDL-C, low-density lipoprotein cholesterol; TG/HDL-C, the ratio of triglyceride to high-density lipoprotein cholesterol; HbA1c, glycated hemoglobin; SBP, systolic blood pressure; DBP, diastolic blood pressure; FBG, Fasting Blood Glucose; hsCRP, high-sensitivity C-reactive protein;2hCP,2hC-peptide;FCP,fasting C-peptide (FCP).

### 6.3 Both groups of patients used China-PAR model(the Prediction for ASCVD Risk in China) for risk prediction of atherosclerotic cardiovascular disease, evaluating the 10-year and lifetime CVD risk

The results are shown in **Table 4**. Both groups showed a decrease in 10-year cardiovascular disease (CVD) risk after the intervention, with the W group experiencing a significant reduction in lifelong CVD risk, while the U group showed less noticeable improvement.

**Table 4:** Comparison of CVD Risk Assessment Between the Two Groups Before and After Intervention in China PAR Model.

		10-year CVD risk			lifetime CVD risk		
		$\bar{x}$ ± s (%)	*t-*value	*P*-Value	$\bar{x}$ ± s (%)	*t-*value	*P*-Value
Group W	Before Intervention	13.47 ± 6.34	-2.34	0.0222^*^	35.55 ± 14.01	-2.46	0.0208^*^
	After Intervention	12.43 ± 6.22			30.33 ± 15.23		
Group U	Before Intervention	13.79 ± 7.46	-2.22	0.0331^*^	33.88 ± 11.91	-1.36	0.1907
	After Intervention	12.45 ± 6.52			31.65 ± 9.98		

## 7 Discussion

In this investigation, the intervention group experienced a notable substitution of their primary dietary sources with a grain-based meal. Upon preliminary computation, the cumulative daily energy contribution furnished by the grain-based regimen within Group W approximates 646 kcal, wherein carbohydrates comprise 70% of the energy allotment. This equates to a comprehensive dietary fiber content of 23.76 g/day, encompassing 8.97 g/day of soluble dietary fiber and 14.79 g/day of insoluble dietary fiber. This estimation encompasses the supplementary intake derived from vegetables, legumes, and fruits. The approximated proportion of daily energy intake sourced from carbohydrates conveniently adheres to the range of 50% to 65%.The results elicited from this approach showcased marked decrease in several crucial parameters, encompassing BMI, WHR, FBG, HbA1c, TC, LDL-C, and hsCRP, vis-à-vis the conventional dietary management group. A meticulous analysis of the nutritional composition characterizing this grain-based dietary regimen holds promise for illuminating its influence on metabolic constituents and conferring cardiovascular benefits.

To commence, the grain-based meal exhibits a notable elevation in dietary fiber content, a component that remains impervious to digestion and showcases robust water-absorption attributes. Upon ingestion, dietary fiber interacts with water, expanding in volume and eliciting a sensation of satiety, thus curbing food intake (17). This decline in food consumption leads to a commensurate reduction in overall energy intake, consequently fostering a favourable milieu for weight reduction.

Dietary fiber and resistant starch, in their incompletely hydrolyzed state within the small intestine, undergo subsequent breakdown by the colonic microbiota. A pivotal outcome of this fermentation process is the generation of short-chain fatty acids (SCFAs), encompassing butyrate, propionate, and acetate. SCFAs are markedly associated with mitigating the impact of T2DM (18). Numerous investigations have underscored the propensity of butyrate salts to diminish appetite and weight by interacting with and activating G-protein-coupled free fatty acid receptors (FFAR) within intestinal enteroendocrine cells (19). This activation instigates the release of glucagon-like peptide-1 (GLP-1) and peptide YY (PYY). GLP-1 augments insulin secretion while inhibiting glucagon release, whereas PYY curbs appetite and retards gastric emptying (20). Furthermore, butyrate occupies a substantial role in modulating the expression of genes pivotal to adipocyte differentiation. Specifically, it orchestrates the augmentation of the messenger ribonucleic acid of Sterol Regulatory Element-Binding Protein-1c (SREBP-1c), a cardinal regulator of adipogenesis and *de novo* fatty acid synthesis. Additionally, it elevates the expression of key adipocyte differentiation markers, including the peroxisome proliferator-activated receptor γ (PPARγ) and CCAAT/enhancer-binding protein alpha (C/EBPα), within adipocytes (21, 22).The soluble dietary fibers used in this study, such as oligofructose and oligogalactose, can lead to higher abundance of bifidobacteria and lactobacilli, as well as increased fecal butyrate levels (23, 24).

The ascendancy of insoluble dietary fiber prompts heightened excretion of bile acids, while its soluble counterpart dissolves within gastrointestinal fluids to engender a gel-like consistency that impedes fat absorption (25). Simultaneously, the trajectory of weight loss precipitates a decrement in plasma free fatty acid (FFA) concentrations (26, 27). This curtailment in free fatty acid levels consequently instigates a reduction in hepatic gluconeogenesis. Further alterations materialize in the form of amplified the phosphatidylinositol-3-kinase (PI3K) activity, pertaining to insulin receptor substrate-1 (IRS-1), and augmented type 4 glucose transporter (GLUT-4) translocation to the muscle surface, culminating in an augmented glucose uptake that serves to alleviate insulin resistance. This cascade serves to diminish pancreatic β-cell vulnerability to lipotoxicity, thus forestalling the deterioration of pancreatic function (28). Plasma FFA reduction can decrease cholesterol ester and triglyceride (TG) synthesis, reduce HDL clearance, and increase its concentration (28). In this study, the intervention group showed a significant decrease in TG/HDL-C levels and an increase in 2hC-peptide/fasting C-peptide levels compared to before the intervention, with significant statistical differences compared to the control group, suggesting that this grain-based nutritional meal is beneficial for improving insulin resistance. The inclusion of dietary fiber can restore the vigour of the phosphoinositide 3-kinase/serine kinase pathway, in turn normalizing serum leptin levels. This normalization cascade culminates in an amelioration of insulin resistance and an augmentation of insulin sensitivity (29).

Chen et al.’s exploration yielded the noteworthy discovery of a substantial reduction in the levels of serum inflammatory chemokines (IL-1β, IL-6) among patients grappling with T2DM subsequent to high-fiber diet intervention (30). Within the present study, the W group exhibited a palpable decline in hsCRP levels relative to the baseline, thus unveiling a statistical distinction relative to the control group. Notably, hsCRP stands as a pivotal risk indicator for untoward cardiovascular consequences within the context of coronary artery disease (31). An assemblage of evidence advances the proposition that in the realm of coronary artery disease patients following guideline-directed medical therapy, the embrace of a vegetarian diet can evoke a reduction in hsCRP levels (32). This outcome underscores the anti-inflammatory potential of the cereal-based nutritional meal, a facet that bears significant import within the context of coronary artery disease.

WC persists as an autonomous risk determinant impinging upon cardiovascular afflictions and all-cause mortality (33). Simultaneously, WHR assumes the mantle of an efficacious indicator for evaluating central obesity. In accordance with the benchmarks stipulated by the World Health Organization, a WHR surpassing 0.90 for males and 0.85 for females flags the advent of central obesity (34). A clinical trial was conducted on 64 overweight and obese adolescents. Half of them were required to supplement daily with chitosan. Chitosan is a dietary fiber. After 12 weeks of follow-up, chitosan supplementation had greater improvement in BMI and WC compared with the placebo group. Differences were significant(*P*< 0.05) (35). Within the ambit of this study’s findings, both the intervention and control groups evinced post-treatment reductions in WC relative to the baseline. Nevertheless, no substantive statistical disparity surfaced between the two groups on this parameter. However, a conspicuous advantage manifested within the change in WHR within the intervention group as compared to the control group. This variance could potentially be ascribed to the demographic composition disparity across the groups, with the control group skewing slightly towards males while the intervention group encompassed a higher proportion of females. Gender exerts a discernible influence upon body fat distribution. Furthermore, a noteworthy facet arises from the recognition that the sole reliance on WC measurements might inadvertently introduce measurement imprecisions. In this regard, the employment of the WHR proves instrumental in mitigating the repercussions of measurement discrepancies. Conclusively, it is believed that grain-based meals have a certain role in improving central obesity.

Noteworthy evidence underscores that tailored dietary patterns, typified by the Dietary Approaches to Stop Hypertension (DASH) diet, when synergistically married with other lifestyle modifications like physical exercise and weight reduction, can furnish efficacious reductions in blood pressure (36). Within the compass of this study, both the intervention and control cohorts exhibited a decline in blood pressure levels post-treatment vis-à-vis the baseline, yet without the emergence of a marked statistical distinction between the groups. This deficiency in statistical significance in inter-group juxtaposition might be ascribed to parallel interventions in sodium salt consumption. Moreover, the mean age bracket of the study populace approximated 60 years, thereby propelling age-related vascular degeneration to manifest as an immutable trigger, delineating a state of decreased responsiveness to blood pressure-lowering interventions akin to the DASH diet (37). Further complexity arises from the recognition that around 40% of patients within both groups had a history of hypertension, a backdrop that substantiates a more accentuated vascular impairment in comparison to their counterparts without such a history. This suggests a noticeable impact of vascular senescence upon the outcomes of interventions (37).

The Mediterranean diet, revered for its composition abundant in β-carotene, vitamin C, vitamin E, natural folate, flavonoids, selenium, and other essential minerals, plays a crucial role. The antioxidative attributes inherent to these constituents engender a mitigation of acute cardiac afflictions. Indicative evidence highlights the capacity of carotenoids to protract the progression of atherosclerotic plaques (38). Included in this cereal-based meal is an array of constituents, encompassing lentils, red beans, soybeans, corn, red dates, goji berries, lotus seeds, and peanuts. Among these, legumes serve as significant sources of unsaturated fatty acids, red dates are rich in vitamin C, while goji berries are endowed with a profusion of goji polysaccharides, β-carotene, vitamin E, selenium, and flavonoids. Together, they possess significant antioxidative properties.

This study utilized the China-PAR risk assessment model to evaluate changes in 10-year and lifelong CVD risk before and after intervention in both groups, offering a clearer indication of the differences in cardiovascular benefits. The results showed a decrease in 10-year CVD risk for both groups after intervention, with a significant reduction in lifetime CVD risk for the W group, while the reduction in the U group was not statistically significant. This study supports the beneficial effects of the cereal-based nutritional intervention on cardiovascular disease.

The strengths of this study include a placebo control and a randomized design. However, some limitations should also be acknowledged. Firstly, it was not a very large sample study, but rather a study with a small sample size conducted at a single center. The gender composition ratio between the two groups and the use of hypoglycemic drugs were not completely balanced. We hope to conduct a larger study with a larger sample size in multiple centers in the future. Secondly, the follow-up period for this study was relatively short, and there may have been some imbalance in the use of treatment medications between the two groups, which could have led to a lack of significant intergroup differences in certain cardiovascular risk factors at the end of the follow-up. Gong Q et al. conducted a 30-year follow-up study and found that lifestyle interventions can reduce the risk of cardiovascular events in individuals with impaired glucose tolerance (IGT) (39). Therefore, the current presentation of some negative results does not necessarily mean that there is no significant difference, but perhaps our observation time is not sufficient.

## 8 Conclusions

In conclusion, this study demonstrates the beneficial effects of a high-fiber grain-based meal on weight, body fat distribution, various metabolic indicators, and cardiovascular risk in patients with type 2 diabetes. Embracing a backdrop of myriad targets and minimal untoward effects, natural food therapy has emerged as an important area of study for improving cardiovascular health (40). Encompassing a stratum of society caught up in the fast-paced nature of modern life, notably the working professionals, the rise in diabetes among younger people has coincided with an increase in fast food consumption. There is a need for effective dietary interventions for this group. Within this spectrum, cereal-based nutritional meals, offering both convenience and multiple cardiovascular benefits, have the potential to be a practical approach in advocating for diabetes dietary management.

## Data availability statement

The raw data supporting the conclusions of this article will be made available by the authors, without undue reservation.

## Ethics statement

The studies involving humans were approved by Ethics Committee of Qidong People’s Hospital. The studies were conducted in accordance with the local legislation and institutional requirements. The participants provided their written informed consent to participate in this study.

## Author contributions

XL: Writing – original draft. YS: Writing – review & editing. DW: Investigation, Data curation. WN: Formal Analysis. NZ: Investigation. XY: Investigation.

## References

[1] Ji L Hu D Pan C Weng J Huo Y Ma C. Primacy of the 3B approach to control risk factors for cardiovascular disease in type 2 diabetes patients. Am J Med (2013) 126(10):925.e11–22. doi: 10.1016/j.amjmed.2013.02.035 23810406

[2] Rawshani A Rawshani A Franzén S Sattar N Eliasson B Svensson AM. Risk factors, mortality, and cardiovascular outcomes in patients with type 2 diabetes. N Engl J Med (2018) 379(7):633–44. doi: 10.1056/NEJMoa1800256 30110583

[3] Gaede P Lund–Andersen H Parving HH Pedersen O. Effect of a multifactorial intervention on mortality in type 2 diabetes. N Engl J Med (2008) 358(6):580–91. doi: 10.1056/NEJMoa0706245 18256393

[4] Zheng Y Lin Y Xu X. Dietary structure investigation and analysis of overweight and obese type 2 diabetes patients. Chin J Diab (2019) 11(11):742–6. doi: 10.3760/cma.j.issn.1674-5809.2019.11.009

[5] Gershuni VM Yan SL Medici V. Nutritional ketosis for weight management and reversal of metabolic syndrome. Curr Nutr Rep (2018) 7(3):97–106. doi: 10.1007/s13668-018-0235-0 30128963 PMC6472268

[6] National Health Commission of the People’s Republic of China. WS/T 578.1-2017 Reference Intake of Nutrients for Chinese Residents. Beijing: China Standard Press (2017).

[7] Seidelmann SB Claggett B Cheng S Henglin M Shah A Steffen LM. Dietary carbohydrate intake and mortality: a prospective cohort study and meta–analysis. Lancet Public Health (2018) 3(9):e419–28. doi: 10.1016/S2468-2667(18)30135-X PMC6339822 30122560

[8] Reynolds A Mann J Cummings J Winter N Mete E Te Morenga L. Carbohydrate quality and human health: a series of systematic reviews and meta–analyses. Lancet (2019) 393(10170):434–45. doi: 10.1016/S0140-6736(18)31809-9 30638909

[9] Evert AB Dennison M Gardner CD Garvey WT Lau KHK MacLeod J. Nutrition therapy for adults with diabetes or prediabetes: a consensus report. Diabetes Care (2019) 42(5):731–54. doi: 10.2337/dci19-0014 PMC7011201 31000505

[10] Conroy RM Pyörälä K Fitzgerald AP Sans S Menotti A De Backer G. Estimation of ten-year risk of fatal cardiovascular disease in Europe: the SCORE project. Eur Heart J (2003) 24(11):987–1003. doi: 10.1016/s0195-668x(03)00114-3 12788299

[11] Hippisley-Cox J Coupland C Vinogradova Y Robson J May M Brindle P. Derivation and validation of QRISK, a new cardiovascular disease risk score for the United Kingdom: prospective open cohort study. BMJ (2007) 335(7611):136. doi: 10.1136/bmj.39261.471806.55 17615182 PMC1925200

[12] Hippisley-Cox J Coupland C Robson J Brindle P. Derivation, validation, and evaluation of a new QRISK model to estimate lifetime risk of cardiovascular disease: cohort study using QResearch database. BMJ (2010) 341:c6624. doi: 10.1136/bmj.c6624 21148212 PMC2999889

[13] Goff DC Jr Lloyd-Jones DM Bennett G Coady S D'Agostino RB Sr Gibbons R. 2013 ACC/AHA guideline on the assessment of cardiovascular risk: a report of the american college of cardiology/american heart association task force on practice guidelines. J Am Coll Cardiol (2014) 63(25 Pt B):2935–59. doi: 10.1016/j.jacc.2013.11.005 PMC4700825 24239921

[14] Yang X Li J Hu D Chen J Li Y Huang J. Predicting the 10-year risks of atherosclerotic cardiovascular disease in Chinese population: the China-PAR project (prediction for ASCVD risk in China). Circulation (2016) 134(19):1430–40. doi: 10.1161/CIRCULATIONAHA.116.022367 27682885

[15] Li S Lin G Chen J Chen Z Xu F Zhu F. The effect of periodic ketogenic diet on newly diagnosed overweight or obese patients with type 2 diabetes. BMC Endocr Disord (2022) 22(1):34. doi: 10.1186/s12902-022-00947-2 35115003 PMC8811985

[16] Zhao L Zhang F Ding X Wu G Lam YY Wang X. Gut bacteria selectively promoted by dietary fibers alleviate type 2 diabetes. Science (2018) 359(6380):1151–6. doi: 10.1126/science.aao5774 29590046

[17] Chinese Nutrition Society Diabetes Nutrition Workgroup. Chinese dietary guidelines for type 2 diabetes and interpretation. Acta Nutrimenta Sinica (2017) 39(6):521–9. doi: 10.3969/j.issn.0512-7955.2017.06.002

[18] Dalile B Van Oudenhove L Vervliet B Verbeke K. The role of short-chain fatty acids in microbiota-gut-brain communication. Nat Rev Gastroenterol Hepatol (2019) 16(8):461–78. doi: 10.1038/s41575-019-0157-3 31123355

[19] Hara T Kimura I Inoue D Ichimura A Hirasawa A. Free fatty acid receptors and their role in regulation of energy metabolism. Rev Physiol Biochem Pharmacol (2013) 164:77–116. doi: 10.1007/112_2013_13 23625068

[20] Steinert RE Feinle-Bisset C Asarian L Horowitz M Beglinger C Geary N. Ghrelin, CCK, GLP-1, and PYY (3–36): Secretory controls and physiological roles in eating and glycemia in health, obesity, and after RYGB. Physiol Rev (2017) 97:411–63. doi: 10.1152/physrev.00031.2014 PMC6151490 28003328

[21] Yan H Ajuwon KM. Mechanism of butyrate stimulation of triglyceride storage and adipokine expression during adipogenic differentiation of porcine stromovascular cells. PloS One (2015) 10(12):e0145940. doi: 10.1371/journal.pone.0145940 26713737 PMC4694642

[22] Hafidi ME Buelna-Chontal M Sánchez-Muñoz F Carbó R. Adipogenesis: A necessary but harmful strategy. Int J Mol Sci (2019) 20:3657. doi: 10.3390/ijms20153657 31357412 PMC6696444

[23] Nicolucci AC Hume MP Martínez I Mayengbam S Walter J Reimer RA. Prebiotics reduce body fat and alter intestinal microbiota in children who are overweight or with obesity. Gastroenterology (2017) 153(3):711–22. doi: 10.1053/j.gastro.2017.05.055 28596023

[24] So D Whelan K Rossi M Morrison M Holtmann G Kelly JT. Dietary fiber intervention on gut microbiota composition in healthy adults: a systematic review and meta-analysis. Am J Clin Nutr (2018) 107(6):965–83. doi: 10.1093/ajcn/nqy041 29757343

[25] Surampudi P Enkhmaa B Anuurad E Berglund L. Lipid lowering with soluble dietary fiber. Curr Atheroscler Rep (2016) 18(12):75. doi: 10.1007/s11883-016-0624-z 27807734

[26] Chearskul S Delbridge E Shulkes A Proietto J Kriketos A. Effect of weight loss and ketosis on postprandial cholecystokinin and free fatty acid concentrations. Am J Clin Nutr (2008) 87(5):1238–46. doi: 10.1093/ajcn/87.5.1238 18469245

[27] Varady KA Dam VT Klempel MC Horne M Cruz R Kroeger CM. Effects of weight loss via high fat vs. low fat alternate day fasting diets on free fatty acid profiles. Sci Rep (2015) 5:7561. doi: 10.1038/srep07561 25557754 PMC5378987

[28] Fahed G Aoun L Bou Zerdan M Allam S Bou Zerdan M Bouferraa Y. Metabolic syndrome: updates on pathophysiology and management in 2021. Int J Mol Sci (2022) 23(2):786. doi: 10.3390/ijms23020786 35054972 PMC8775991

[29] Chen J Raymond K. Beta-glucans in the treatment of diabetes and associated cardiovascular risks. Vasc Health Risk Manage (2008) 4(6):1265–72. doi: 10.2147/vhrm.s3803 PMC2663451 19337540

[30] Chen L Liu B Ren L Du H Fei C Qian C. High-fiber diet ameliorates gut microbiota, serum metabolism and emotional mood in type 2 diabetes patients. Front Cell Infect Microbiol (2023) 13:1069954. doi: 10.3389/fcimb.2023.1069954 36794003 PMC9922700

[31] Shah B Newman JD Woolf K Ganguzza L Guo Y Allen N. Anti-inflammatory effects of a vegan diet versus the american heart association-recommended diet in coronary artery disease trial. J Am Heart Assoc (2018) 7(23):e011367. doi: 10.1161/JAHA.118.011367 30571591 PMC6405545

[32] Trautwein EA McKay S. The role of specific components of a plant-based diet in management of dyslipidemia and the impact on cardiovascular risk. Nutrients (2020) 12(9):2671. doi: 10.3390/nu12092671 32883047 PMC7551487

[33] Ross R Neeland IJ Yamashita S Shai I Seidell J Magni P. Waist circumference as a vital sign in clinical practice: A Consensus Statement from the IAS and ICCR Working Group on Visceral Obesity. Nat Rev Endocrinol (2020) 16:177–89. doi: 10.1038/s41574-019-0310-7 PMC7027970 32020062

[34] Minetto MA Pietrobelli A Busso C Bennett JP Ferraris A Shepherd JA. Digital anthropometry for body circumference measurements: European phenotypic variations throughout the decades. J Pers Med (2022) 12(6):906. doi: 10.3390/jpm12060906 35743690 PMC9224732

[35] Fatahi S Sayyari AA Salehi M Safa M Sohouli M Shidfar F. The effects of chitosan supplementation on anthropometric indicators of obesity, lipid and glycemic profiles, and appetite-regulated hormones in adolescents with overweight or obesity: a randomized, double-blind clinical trial. BMC Pediatr (2022) 22(1):527. doi: 10.1186/s12887-022-03590-x 36064382 PMC9442917

[36] Filippou CD Tsioufis CP Thomopoulos CG Mihas CC Dimitriadis KS Sotiropoulou LI. Dietary approaches to stop hypertension (DASH) diet and blood pressure reduction in adults with and without hypertension: A systematic review and meta-analysis of randomized controlled trials. Adv Nutr (2020) 11(5):1150–60. doi: 10.1093/advances/nmaa041 PMC7490167 32330233

[37] Carey RM Muntner P Bosworth HB Whelton PK. Prevention and control of hypertension: JACC health promotion series. J Am Coll Cardiol (2018) 72(11):1278–93. doi: 10.1016/j.jacc.2018.07.008 PMC6481176 30190007

[38] Tuttolomondo A Simonetta I Daidone M Mogavero A Ortello A Pinto A. Metabolic and vascular effect of the mediterranean diet. Int J Mol Sci (2019) 20(19):4716. doi: 10.3390/ijms20194716 31547615 PMC6801699

[39] Gong Q Zhang P Wang J Ma J An Y Chen Y. Morbidity and mortality after lifestyle intervention for people with impaired glucose tolerance: 30-year results of the Da Qing Diabetes Prevention Outcome Study. Lancet Diabetes Endocrinol (2019) 7(6):452–61. doi: 10.1016/S2213-8587(19)30093-2 PMC8172050 31036503

[40] Hao P Jiang F Cheng J Ma L Zhang Y Zhao Y. Traditional Chinese medicine for cardiovascular disease: evidence and potential mechanisms. J Am Coll Cardiol (2017) 69(24):2952–66. doi: 10.1016/j.jacc.2017.04.041 28619197

